# Mental Health Condition among University Students of Bangladesh during the Critical COVID-19 Period

**DOI:** 10.3390/jcm11154617

**Published:** 2022-08-08

**Authors:** Md Mostafizur Rahman, Asikunnaby, Saadmaan Jubayer Khan, Anuva Arony, Zahid Al Mamun, Nawwar Fatima Procheta, Mohammed Sadman Sakib, Komal Raj Aryal, Farzana Rahman, Abu Reza Md. Towfiqul Islam

**Affiliations:** 1 Department of Disaster Management and Resilience, Faculty of Arts and Social Sciences, Bangladesh University of Professionals, Mirpur Cantonment, Dhaka 1216, Bangladesh; 2 Department of Disaster and Human Security Management, Faculty of Arts and Social Sciences, Bangladesh University of Professionals, Mirpur Cantonment, Dhaka 1216, Bangladesh; 3 Crisis and Disaster Management, Aston Business School, Aston University, Birmingham B4 7ET, UK; 4 Department of Computer Science and Engineering, Independent University, Dhaka 1212, Bangladesh; 5 Department of Disaster Management, Begum Rokeya University, Rangpur 5400, Bangladesh

**Keywords:** COVID-19, university students, mental health, stress, lockdown, tertiary education

## Abstract

Bangladesh’s education sector has been in a state of flux since COVID-19. During the pandemic, all university campuses were closed. There was a mental health issue among the students. This study aims to examine the mental health condition and the determinants that contribute to adverse mental health conditions among university students of Bangladesh. A survey was performed online among university students in Bangladesh, in mid-June 2020 when averaging 3345 affected cases of the population daily. The convenience sampling technique was used and the survey gathered data from 365 university students. The relationship between general information and Depression, Anxiety, and Stress Scale 21 (DASS-21) subscales of university students was determined. The questionnaire was administered to respondents during the pandemic, which ensured fast replies. Linear regression models were used for statistical analysis. University students indicated normal levels of depression (30.41%), anxiety (43.29%), and stress (47.40%). However, a disproportionate number of extremely depressed, anxious, and stressed university students suggested a mental health status of concern. There were significant connections between the individual’s opinion of social satisfaction, mental health concerns, and the present location’s safety with an undesirable mental health condition. Female students were shown to be much more anxious and stressed than male students. Capital Dhaka city students were more depressed and anxious than students outside of Dhaka. Financial and psychological support for students may help mitigate the psychological impact. Authorities should make effective efforts to reduce mental health problems among these students. This research may aid organizations, health care providers, and social workers in their attempts to prepare for and respond to pandemics.

## 1. Introduction

Coronavirus disease 2019 (COVID-19)’s widespread influence has placed several nations in perilous circumstances. The World Health Organization (WHO) classified this infectious disease as a pandemic in December 2019 due to its fast spread from Wuhan, China [1,2,3] to Europe, the United States, Brazil, and South-East Asia [4,5]. According to the Johns Hopkins University, the overall number of COVID-19 cases worldwide through the mid of December 2020 was 75,779,478, and the total number of deaths was 1,677,351 [5]. Over 200 countries have already felt the devastation of a pandemic [6]. This contagion has developed into a long-term catastrophe, wreaking havoc on society, economies, psychological well-being, and governance [1,7,8,9,10,11,12,13]. 

Countries have previously witnessed the psychological impact of infectious diseases, and the global community has now undergone a similar experience due to COVID-19 [14,15,16,17]. During and after pandemics and other calamities, it has been observed that the psychological impact on both directly and indirectly impacted people is ignored [18,19,20,21]. The psychological status of these individuals may not be addressed in the same manner as their physical injuries. However, calamity and its impact on mental health have a close link that makes for an essential research subject [18,20,21]. In the past, infectious diseases have had psychological effects on several communities. 

COVID-19 has been termed “Coronaphobia”, requiring essential steps to mitigate the psychological impact [17,22]. Not only is fear of infection a component in the psychological impact of COVID-19, but so are issues such as long-term lockdown, quarantine time, socio-economic disruption, dread of uncertainty, and adjusting to a new normal scenario. Study has indicated the prevalence of depression, anxiety, and stress during the COVID-19 period in Bangladesh [7]. Researcher also found that the public university students of Bangladesh had depression, anxiety, and stress [11]. Vulnerable people had severe mental health conditions in Bangladesh due to COVID-19 [23]. In the event of a severe acute syndrome (SARS) outbreak, a study found a link between prolonged quarantine and the incidence of adverse mental health [24]. Mental health conditions can also be affected due to the pandemic [7]. The facilitators in this situation have encountered problems reaching out to the psychologically vulnerable population. It is worth noting that the long-term psychological impact has yet to be determined; in fact, many developing countries consider it a delicate subject to discuss [18,21].

Along with socio-economic sectors, the education sector has been impacted by the COVID-19 pandemic, with governments directed to shut educational institutions to contain the outbreak [25,26]. Numerous institutions have begun offering online programs to help students continue their studies. However, these online academic activities were novel to institutions, and poorer students have struggled academically, particularly in developing countries where many locations may lack adequate internet access. Not only have university students been infected with COVID-19; they have also struggled to sustain their academic activities. Due to COVID-19, they have become the most vulnerable groups [7,8,25,26,27]. 

As of mid-December 2020, Bangladesh had documented 498,923 COVID-19 cases, with over 7000 deaths being officially confirmed, while neighbouring India became the world’s second worst-hit country [5]. On 26 March 2020, Bangladesh declared lockdown [28]. From June 2020, the government saw a dramatic increase in COVID-19 cases, and the fatality rate increased significantly following the lifting of the lockdown on 31 May 2020. Dhaka has been one of the hardest-hit places by COVID-19 [29]. Psychological consequences of this pandemic have been noticeable among the majority of Bangladeshis; there has even been one suicidal case due to anxiety of COVID-19 infection [7,15,16]. University students are also victims of COVID-19. In a developing country such as Bangladesh, university students sometimes have additional duties such as supporting family members, assisting communities in certain regions, and participating in social activities. They are unique individuals who can contribute to the country’s development. Families often wait for their university-enrolled relatives to complete their studies within a predetermined time frame for assisting their respective families. Bangladesh was formerly faced with session congestion at the university level of education [30], where universities temporarily suspended academic operations, which has been very rare in recent years. Typically, it has occurred as a result of political instability and bloodshed. COVID-19, on the other hand, has reintroduced this difficult position. Universities have already struggled to finish their regularly scheduled academic sessions due to irregular academic activity during the COVID-19 pandemic. Several institutions have started academic operations online in response to the University Grants Commission (UGC) of Bangladesh’s directive [31]. However, these online activities are novel for Bangladeshi universities. Not only that students, particularly those living in rural regions, have faced a degraded internet network but also worry of infection, disrupted academic activities, fear of career prospects, and other associated variables may have placed these university students in an unprecedented scenario, exacerbating the group’s adverse mental health state. 

This study concentrated on the critical COVID-19 period, from 7 June to 29 June during which the country observed COVID-19 cases averaging 3345 person/day [32]. We applied Depression, Anxiety, and Stress Scale-21 (DASS 21) [33,34]. Using DASS 21, numerous research studies have been conducted since the emergence of the COVID-19 pandemic to explore the prevalence of mental health issues and to identify its determinants in various groups [7,11,35,36,37]. One study, performed in seven middle-income countries of Asia, observed that Thailand had the highest scores on the DASS 21 scale [36]. In a subsequent longitudinal research done in 190 Chinese towns, the occurrence of symptoms including fever with cough or trouble breathing and recent quarantine was substantially related with DASS 21 scores for stress, anxiety, and depression [37]. Study, conducted in Spain, showed that physical activity could reduce the DASS 21 score [38]. Studies were also conducted to evaluate the impact of COVID-19 among university students of Bangladesh by using DASS 21 [23,39]. However, from our understanding there was not enough input regarding mental health conditions among university students of Bangladesh considering the peak of the COVID-19 period. The purpose of our study was to evaluate the mental health condition among university students of Bangladesh during that critical period of COVID-19. We also assessed the relationship between the students’ detailed information (socio-demographic data, academic data, and university support) during that period and their mental health condition. Thus, the research questions for this study were as follows:

What were the overall mental health conditions among university students of Bangladesh during the peak of COVID-19 cases?

What were the determinants of depression, anxiety, and stress among these students during that period?

How did the universities support these students to reduce mental health conditions due to the increased COVID-19 cases? 

## 2. Materials and Methods

### 2.1. Research Design and Ethical Issues

This study was performed using an online-based self-administered survey in Bangladesh during the COVID-19 period. Except for certain online activities, all university campuses remained closed. The research region was separated into two sections: the most COVID-19 impacted area Dhaka city, and outside the city, highlighting the existing university students. Participating university students’ mental health status was assessed to determine links between their demographic information, academic profile, socio-academic situation, and university assistance. This study was conducted as part of an approved research project (BUP REC-343/2019) by the Research Ethics Committee of Bangladesh University of Professionals, Dhaka, Bangladesh., which adhered to all ethical standards. The cover page of the questionnaire stated the purpose of the study. It was also assured that all replies gathered would stay secret, as they were solely to be used for research purposes. Online consents were also taken. There was no incentive offered to the participants. 

### 2.2. Study Tool

Relevant studies on mental health conditions were reviewed [17,18,33,40,41,42]. Discussion with experts such as the university psychological counselor was also conducted. In addition, a pilot survey was conducted among a small group of university students. The final online questionnaire consisted of five sections; university students’ demographic information (age, gender, current location, and if they lived with their family during the survey), academic profile (type of university (government funded or private), location of the university (in capital Dhaka city or outside the city), students’ current academic year, and major of study), socio-academic condition (if they were concerned about mental health during that period, their confidence in the living place regarding COVID-19 prevention, current social life, academic performance, their concern about study during that period, and concern regarding family member’s earning), the university supports for the students (if there was any COVID-19 related subject in their curriculum, financial/mental support from the university, and online class to continue regular academic activities), and widely used DASS 21 questions [11,33,41,42,43]. Both English and verified Bengali translated versions of DASS 21 questions [33,41] were administered among university students of Bangladesh. The first DASS consisted of 42 items on a four-point Likert-scale to evaluate the negative emotional states of depression, anxiety, and stress, with 14 questions for each subscale. In 2005, Henry and Crawford created and validated a shorter version of the DASS, the DASS 21 [34], where each sub-scale of depression, anxiety, and stress consisted of seven items. The presence and absence of depression, anxiety, and stress were evaluated using the total of the scores for each sub-seven scale’s items. The existence of depression was indicated by a sum of scores ≥10, and where it was ≥8 and ≥15 for anxiety and stress, respectively (Table 1). For reliability, we calculated the overall Cronbach’s alpha (α) of the DASS 21 which was 0.94, indicating excellent internal consistency [44], while the Cronbach’s α of each sub-scale was α = 0.89 (depression), α = 0.81 (anxiety), and α = 0.88 (stress), respectively. 

### 2.3. Data Collection

The survey was performed in mid-June 2020 when on average 3345 people were being affected daily [32]. The data gathering process was designed to ascertain quick replies during the peak of COVID-19 instances [29]. The questionnaire was administered to the respondents during this period. Certain universities conducted all academic activities solely online. Due to the continuing pandemic, a convenience sampling technique was considered for data collection. This strategy has been effectively employed in earlier COVID-19-related studies [7,9]. The authors initially instructed their department’s students through the Zoom live stream on the importance and objective of the study. In addition, ethical issues involved in the study, the process of distributing the URL and the probable challenges and questions along with the solutions which might be required by the participants, were also described. Together with the authors, the studying university students distributed the URL to their peers. The online questionnaire was sent to other university students using social media platforms like Facebook and WhatsApp. 

### 2.4. Data Management and Analysis

All data management and data analyses were conducted by ‘Python (version 2.7; Beaverton, OR, USA), RStudio (version 1.2.5042; Boston, MA, USA), and ‘R’ programming language (version 3.6.3; Vienna, Austria) [45,46,47] with 95% confidence interval (95% CI). Descriptive statistics were measured where appropriate. The severity labels of depression, anxiety, and stress were measured following the recommended cut-off scores for conventional severity labels shown in Table 1 [33]. The association between demographic profile, academic profile, socio-academic condition, the university supports for students, and DASS 21 subscales were analyzed through linear regression models. Previous relevant studies have also successfully applied linear regression models [7,11]. Our objective was to identify the individual predictors of their mental health conditions. 

## 3. Results

Table 2 presents the frequency and percentages of depression, anxiety, and stress labels among university students of Bangladesh. The respondents were labeled depending on the score range of DASS-21 (Table 1) [33], representing the frequency and percentages of the respondents who scored in the labeled range. The majority of the students demonstrated a normal depression label (30.41%) However, some were also moderately (23.01%) and extremely depressed (23.01%). In the case of the anxiety label, the majority of the students showed normal anxiety (43.29%), whereas many of them also demonstrated moderate anxiety (22.47%) and extremely severe anxiety (20.55%). The majority of the university students showed a normal stress label (47.40%) accompanied by moderate (14.79%) and severe (13.42%) stress label during COVID-19.

Table 3 shows the association of university students’ demographic information and academic profile with the DASS 21 subscales. Of the students 49.04% were in the 22 to 25 age group followed by 48.49% in the 18 to 21 age group among 365 respondents, 43.84% male and 56.16% were female; many students were living in the capital Dhaka city (63.84%) during the survey. These students were with their family (98.63%); a large proportion of them were from the government-funded public universities (70.14%) located in Dhaka city (89.59%). The majority of these students were from the undergraduate level with arts and social sciences (38.08%). Male students showed significantly less anxiety (B = −1.97, 95% CI: −3.83; −0.11) and stress (B = −3.85, 95% CI: −6.17; −1.53) compared to female participants; residents from outside of Dhaka showed significantly lower depression (B = −3.54, 95% CI: −6.04; −1.04) and lower anxiety scores (B = −2.28, 95% CI: −4.20; −0.37). Public university students showed higher depression (B = 2.74, 95% CI: 0.11; 5.38), whereas students from the universities outside of Dhaka demonstrated significantly lower depression (B = −5.01, 95% CI: −8.95; −1.07) and lower anxiety (B = −3.24, 95% CI: −6.26; −0.22). In the case of the university year, Masters students were significantly more likely to have stress (B = 4.51, 95% CI: 0.17; 8.86), whereas science and engineering background students were significantly less likely to have stress (B = −3.10, 95% CI: −6.08; −0.12). 

Table 4 presents the association between students’ social and academic conditions and DASS 21 subscales. Many students demonstrated high (55.34%) and moderate (35.89%) concern about mental health during COVID-19. Participants with moderate concerns about their mental health were significantly less likely to have depression (B = −6.23, 95% CI: −8.73; −3.72), anxiety (B = −4.67, 95% CI: −6.59; −2.76), and stress (B = −4.99, 95% CI: −7.41; −2.57), and in addition the students showing low concerns were also significantly less likely to have depression (B = −7.78, 95% CI: −12.03; −3.54), anxiety (B = −6.01, 95% CI: −9.26; −2.77), and stress (B = −8.75, 95% CI: −12.85; −4.65). In the case of students’ confidence about their current residence during COVID-19, the majority indicated that their place was moderately safe (39.45%) and unsafe (29.32%). Students who felt their current living place was very unsafe for COVID-19 showed significant association with higher depression (B = 5.24, 95% CI: 1.37; 9.11), higher anxiety (B = 3.29, 95% CI: 0.32; 6.26), and higher stress (B = 5.55, 95% CI: 1.83; 9.27). In the case of the perception of current social life during COVID-19, the majority of the participants demonstrated low satisfaction (64.38%). Very satisfied students about their social life demonstrated significantly lower depression (B = −10.17, 95% CI: −16.13; −4.22) and lower stress (B = −7.28, 95% CI: −13.07; −1.48); in addition satisfied students also showed significant association with lower depression (B = −5.98, 95% CI: −8.52; −3.43), lower anxiety (B = −3.56, 95% CI: −5.54; −1.57), and lower stress (B = −5.12, 95% CI: −7.59; −2.64). In the case of perception on academic performance, many students showed their satisfaction (53.70%) even during the COVID-19 pandemic. However, many students expressed high concern (58.36%) about the interruption of their regular form of study. Additionally, many students showed low satisfaction level (41.37%) on their academic performances. Satisfied students were significantly less likely to have depression (B = −2.86, 95% CI: −5.35; −0.36). The majority of the participants indicated high (36.44%) and medium (36.44%) concerns about their family member’s earnings during COVID-19, students who had medium concerns demonstrated significant association with lower depression (B = −3.19, 95% CI: −6.01; −0.36). 

Table 5 presents the association between the support from the universities and DASS 21 subscales. The majority of the participated students confirmed (47.95%) that they did not have any subject related to COVID-19 in their university curriculum. The majority of the participants did not receive financial and mental health support (61.64%) from their respective universities. Students who received such support showed significantly less anxiety (B = −2.11; 95% CI: −4.01; −0.22). The majority of these students (84.38%) demonstrated online academic activities.

## 4. Discussion

### 4.1. Overall Mental Health Conditions

This work demonstrates that the study population had extremely severe depression and anxiety during the COVID-19 outbreak. Additionally, it detected extreme stress, which corresponds to prior research findings suggesting a substantial risk of unfavorable mental health prevalence among students [17,43]. Study found that around 38%, 37%, and 25% Bangladeshis showed the prevalence of depression, anxiety, and stress during the COVID-19 period [7]. Mental health condition was also found to be severe in the case of vulnerable people, such as rickshaw pullers in Bangladesh due to COVID-19 [23]. Study found that the students were associated with higher psychological impact during the COVID-19 pandemic [36]. Study performed among public university students of Bangladesh showed that around 38%, 27%, and 18% had depression, severe anxiety, and moderate stress [11]. Subsequently, another study found that 35.2%, 40.3%, and 37.7% of Bangladeshi university students had severe symptoms of prevalence of depression, anxiety, and stress during the COVID-19 pandemic [39]. COVID-19 has already led to uncertainty for university students [48,49]. A study suggested depression and anxiety among Bangladeshi university students during COVID-19, which corroborates the conclusions of this research [11,50]. Furthermore, the increase in COVID-19 instances may have triggered these detrimental mental health conditions. Students at universities may have been fearful about infection [51]. The UGC has previously advised all universities to conduct online academic activities to reduce the disease among students and maintain academic activities [31]. However, given the pandemic’s extended duration, proactive preparation is necessary. Universities should integrate online portals and web-based tools into their curricula to assist students in coping with the negative psychological effects of COVID-19 [17,52]. Moreover, health care providers can intervene via smartphone-based psychoeducation to target this extremely responsive population [17,53]. The government may maintain stringent precautions throughout the period and throughout this period, it should provide physical and psychological assistance to the broader public, especially university students. The findings of a study suggest that authorities should concentrate on effective methods of disseminating unbiased COVID-19 knowledge, instructing proper containment methods, ensuring the availability of essential services/commodities, and providing adequate financial support to reduce mental health issues [37]. Another study found that increasing awareness about a pandemic like COVID-19 and handling the emergency will be difficult if individuals do not recognize the risk of any emergency and do not seek appropriate information [13]. 

### 4.2. Determinants of Depression, Anxiety, and Stress 

This study also examined the relationship between university students’ demographic, social, and academic information with their mental health status throughout COVID-19. Female university students reported higher anxiety and stress levels than their male peers. This finding is consistent with prior research demonstrating that hormonal variables contribute to female candidates experiencing higher depression [54,55]. A national wide study also found that female students were significantly affected by mental health problems during the COVID-19 period [39]. Females typically have difficulties in terms of safety and security during times of crisis in the Bangladeshi context [56]. COVID- 19 has already triggered a global emergency, contributing to female university students’ mental health problems [57].

Dhaka has been one of the most severely affected areas by COVID-19 [26]. The findings revealed that university students outside Dhaka city suffered less depression and anxiety during COVID-19 than those living in Dhaka. Additionally, students from universities located in Dhaka city demonstrated much higher levels of depression and anxiety than students from universities located outside of this megacity. This corroborates with the previous study which showed the prevalence of mental health problems among students living in urban areas [39]. The dramatic increase of COVID-19 cases, notably in Dhaka, may be the cause of these adverse mental health conditions [29]. This study found that the students attending public universities were more likely to experience depression than those attending private universities. It was also found in a previous study that public university students of Bangladesh had mental health issues during the COVID-19 period [11]. Many government-supported public universities did not offer online courses during the current pandemic, although most privately financed universities did. The potential influence of COVID-19 on the academic engagement of public university students may play a role in the development of these adverse mental health conditions. This study established that Master’s students had greater levels of stress. The likely reason is that the Master’s students were awaiting graduation before looking for suitable jobs. Their studies and employment searches were halted due to the COVID-19, which may have contributed to their stress. Additionally, age discrimination is a significant barrier in obtaining government jobs in Bangladesh, which may amplify mental health conditions for Master’s students. Our analysis found that university students with science and engineering backgrounds had less stress. The reasons behind this are outside the scope of this study. However, online education proved challenging for students with a science or engineering background due to the availability of several subjects that required physical laboratory work. One possible explanation for their low-stress levels is the absence of academic pressure during COVID-19. Online education, on the other hand, may have assured their continued education. 

Our findings revealed that many university students were concerned about their mental health, the safety of their current living situation, the satisfaction of their current social life, their academic performance, the interruption of their studies, and the earning potential of their family members during the survey period. Significant associations with DASS 21 subscales were observed by students who were extremely concerned about their mental health. This finding verified the substantial correlation between university students’ perceptions of their mental health and the DASS 21-measured adverse mental health states. Socio-economic status might be a crucial determinant of their mental health [43,58,59,60]. Additionally, this study examined this relationship among the study group. Numerous students expressed dissatisfaction with their existing social lives, yet extremely pleased and satisfied university students reported having less unpleasant mental health conditions. Various mass media outlets reported that the general public disregarded the government’s directive to take precautionary steps. This environment may have contributed to these students’ dread of infection. It may also have disrupted their social and family lives. Students concerned about the earnings of their family members experienced significant depression. The pandemic has put individuals from all sectors of society in a tough position, with economic sectors bearing the brunt of the impact. Numerous employees in Bangladesh lost their employment or it had an adverse effect on their current source of income [61]. 

Additionally, the present study found that many university students encountered extremely risky conditions, demonstrating an adverse mental health status. This conclusion corroborates prior findings (Table 3), which indicated that university students living in the worst-affected COVID-19-affected areas of Dhaka reported a negative mental health state. This study also assessed academic achievement and the effect of COVID-19 on university students’ studies; pleased students had lower levels of depression. This finding shows consistency with a previous study which also showed that dissatisfaction in academic life during COVID-19 period might have been the determinant of mental health problems [39]. Even when certain institutions in Bangladesh began offering online courses, many underprivileged students struggled academically. During the pandemic, suicide behavior was seen among university students engaged in online academic engagement in Bangladesh [62]. Compared to other developed nations, mental health is a delicate topic in the South-Asian continent. Generally, people dislike their mental health status being questioned. However, if organizations could have identified the elements that contribute to mental health problems, they may have been able to intervene to improve the psychological state during COVID-19. Students at universities are one of the most susceptible populations in terms of adverse mental health [17,43]. This study can aid organizations, health care providers, and social workers in understanding the elements that influence university students’ demographic and socio-academic information. These stakeholders should identify the most COVID-19-vulnerable locations and enable psychological support for university students living in those areas. Furthermore, social groups should guarantee that university students have a fulfilling social life, which can help them mentally.

### 4.3. The Support from Universities

A few universities in Bangladesh provided average support for continuing online education, while others have only just started taking online classes. In response to the University Grants Commission (UGC) Bangladesh’s directive, several institutions have started academic operations online [31]. In order to continue online education a few of the universities provided support which was of average quality [63]. The support by the university was considered as a positive impact of wellbeing [64]. The current study analyzed university assistance for their students, including any COVID-19-related courses included in their university curriculum, financial and mental help for impoverished students, and online classes. Our findings revealed that providing financial and psychological support to university students might help mitigate psychological distress, as the results (Table 5) show that the students who received financial and mental assistance reported significantly less anxiety (B = −2.11; 95% CI: −4.01; −0.22) compared to the students who did not receive any financial and mental assistance.

### 4.4. Strengths and Limitations

Due to the situation’s urgency, our study conducted an online survey. Given the continuing pandemic, the most convenient sampling approach was used. The online approach has many advantages as the anonymity of the respondents was maintained; easy access to the data, as it was recorded automatically, made the data processing task efficient. However, students with no internet were not able to participate. Due to the nature of the pandemic and the urgency with which replies had to be collected, a large sample size was not viable. However, this exploratory study may give valuable information to groups seeking to alleviate stress and promote the mental health of university students in Bangladesh. The findings should provide further opportunities for other countries to investigate mental health conditions during the COVID-19 pandemic. 

## 5. Conclusions

COVID-19 responses require a comprehensive strategy in which all sectors collaborate to address the pandemic’s diverse effects. Bangladesh has been severely struck by the COVID-19 pandemic, along with other developed and developing countries. Coronavirus has grown into a phobia in several sectors of the country. The pandemic’s influence has spread beyond the health care sector into the socio-economic and educational sectors. As a result of the long-term impact on those sectors, numerous groups have suffered from mental health problems. However, psychological topics are considered of a delicate nature in Bangladesh, and individuals are unwilling to share their psychological state. This influence may have long-term consequences, and facilitators often struggle to identify psychologically vulnerable groups. Rapid identification based on generic data might aid in this endeavor. The health sector could use socio-economic data to understand this vulnerability better. In the case of university students who are at a high risk of psychological distress during COVID-19, health sector treatments can also take academic information into account. Governments, local governments, and international non-governmental groups can use this data to develop a comprehensive pandemic preparedness and response strategy. In general, our study highlights the significance of severe prevention measures, awareness campaigns, and other supporting activities, such as online academic activities; and social, financial, and mental support to be taken during the fast development of COVID-19 cases and such similar pandemics that may arise. 

## Figures and Tables

**Table 1:** Recommended cut-off scores for conventional severity labels in DASS 21 [33].

Rating	Depression	Anxiety	Stress
Normal	0–9	0–7	0–14
Mild	10–13	8–9	15–18
Moderate	14–20	10–14	19–25
Severe	21–27	15–19	26–33
Extremely Severe	28+	20+	34+

**Table 2:** Depression, anxiety and stress severity labels.

Rating	Depression (*n* (%))	Anxiety (*n* (%))	Stress (*n* (%))
Normal	111 (30.41)	158 (43.29)	173 (47.40)
Mild	53 (14.52)	25 (6.85)	43 (11.78)
Moderate	84 (23.01)	82 (22.47)	54 (14.79)
Severe	33 (9.04)	25 (6.85)	49 (13.42)
Extremely Severe	84 (23.01)	75 (20.55)	46 (12.60)

**Table 3:** Association between demographic and academic profile with DASS 21 subscales.

		Depression			Anxiety			Stress		
Features	*n* (%)	R^2^	β^#^ (95% CI)	*p*	R^2^	β (95% CI)	*p*	R^2^	β (95% CI)	*p*
1. Age										
*a.* *18–21*	177 (48.49)		Reference			Reference			Reference	
*b.* *22–25*	179 (49.04)	0.004	0.90 (−1.56; 3.36)	0.472	0.001	0.39 (−1.50; 2.26)	0.688	0.004	0.73 (−1.64; 3.09)	0.547
*c.* *26–30*	08 (2.19)		3.18 (−5.21; 11.57)	0.456		1.99 (−4.43; 8.41)	0.542		4.46 (−3.62; 12.54)	0.278
*d.* *31–40*	01 (0.27)		−8.32 (−31.59; 14.96)	0.483		1.49 (−16.32; 19.31)	0.869		2.96 (−19.45; 25.37)	0.795
2. Gender										
*a.* *Male*	160 (43.84)	0.002	−1.19 (−3.63; 1.25)	0.339	0.012	−1.97 (−3.83; −0.11) *	0.0376	0.028	−3.85 (−6.17; −1.53) **	0.001
*b.* *Female*	205 (56.16)		Reference			Reference			Reference	
3. Current Location										
*a.* *Dhaka*	233 (63.84)	0.021	Reference		0.015	Reference		0.009	Reference	
*b.* *Outside Dhaka*	132 (36.16)		−3.54 (−6.04; −1.04) **	0.006		−2.28 (−4.20; −0.37) *	0.020		−2.18 (−4.61; 0.24)	0.077
4. Living with family										
*a.* *Yes*	360 (98.63)	0.002	−4.46 (−14.89; 5.98)	0.402	0.005	−5.73 (−13.69; 2.23)	0.158	0.004	−6.59 (−16.62; 3.44)	0.197
*b.* *No*	05 (1.37)		Reference			Reference			Reference	
5. University Type										
*a.* *Public*	256 (70.14)	0.011	2.74 (0.11; 5.38) *	0.041	0.001	0.49 (−1.54; 2.51)	0.637	0.009	(−0.22; 4.87)	0.073
*b.* *Private*	109 (29.86)		Reference			Reference			Reference	
6. University Location										
*a.* *Dhaka*	327 (89.59)	0.017	Reference		0.012	Reference		0.009	Reference	
*b.* *Outside Dhaka*	38 (10.41)		−5.01 (−8.95; −1.07) *	0.013		−3.24 (−6.26; −0.22) *	0.035		−3.61 (−7.42; 0.19)	0.063
7.University Year										
*a.* *First-year*	105 (28.77)	0.011	Reference		0.011	Reference		0.014	Reference	
*b.* *Second-year*	81 (22.19)		−1.64 (5.07; 1.78)	0.345		0.77 (−1.85; 3.39)	0.563		1.17 (−2.12; 4.46)	0.486
*c.* *Third-year*	80 (21.92)		−1.10 (−4.54; 2.33)	0.528		−0.17 (−2.79; 2.46)	0.899		−0.31 (−3.61; 2.99)	0.853
*d.* *Fourth-year*	64 (17.53)		0.72 (−2.95; 4.39)	0.699		2.01 (−0.79; 4.82)	0.159		1.10 (−2.43; 4.62)	0.542
*e.* *Masters*	35 (9.59)		2.68 (−1.83; 7.20)	0.243		2.50 (−0.96; 5.95)	0.156		4.51 (0.17; 8.86) *	0.042
8. Major										
*a.* *Arts and Social Sciences*	139 (38.08)		Reference			Reference			Reference	
*b.* *Business Studies and Economics*	93 (25.48)		1.23 (−1.86; 4.32)	0.436		−1.28 (−3.66; 1.09)	0.289		−1.00 (−3.98; 1.99)	0.511
*c.* *Medical Studies*	30 (8.22)	0.017	−1.66 (−6.31; 2.99)	0.482	0.009	−2.64 (−6.21; 0.92)	0.146	0.012	−1.34 (−5.83; 3.14)	0.556
*d.* *Science and Engineering*	94 (25.75)		−2.65 (−5.73; 0.43)	0.092		−1.54 (−3.91; 0.82)	0.201		−3.10 (−6.08; −0.12) *	0.041
*e.* *Security and Strategic Studies*	9 (2.47)		−3.40 (−11.34; 4.54)	0.401		−1.04 (−7.14; 5.05)	0.736		−0.45 (−8.12; 7.21)	0.907

* *p* < 0.05; ** *p* < 0.01; β^#^ = Beta.

**Table 4:** Association between social and academic conditions with DASS 21 subscales.

		Depression			Anxiety			Stress		
Features	*n* (%)	R^2^	β^#^ (95% CI)	*p*	R^2^	β (95% CI)	*p*	R^2^	β (95% CI)	*p*
1. Concerned about Mental Health										
*a.* *High*	202 (55.34)		Reference			Reference			Reference	
*b.* *Moderate*	131 (35.89)	0.077	−6.23 (−8.73; −3.72) ***	<0.001	0.076	−4.67 (−6.59; −2.76) ***	<0.001	0.07	−4.99 (−7.41; −2.57) ***	<0.001
*c.* *Low*	32 (8.77)		−7.78 (−12.03; −3.54) ***	<0.001		−6.01 (−9.26; −2.77) ***	<0.001		−8.75 (−12.85; −4.65) ***	<0.001
2. Confidence on Current Place for COVID-19										
*a.* *Very Safe*	7 (1.92)		−0.39 (−9.31; 8.53)	0.931		−1.09 (−7.92; 5.75)	0.755		−2.08 (−10.64; 6.49)	0.634
*b.* *Safe*	60 (16.44)		0.01 (−3.53; 3.55)	0.994		0.26 (−2.46; 2.97)	0.853		0.71 (−2.69; 4.11)	0.683
*c.* *Moderately Safe*	144 (39.45)	0.021	Reference		0.015	Reference		0.025	Reference	
*d.* *Unsafe*	107 (29.32)		1.08 (−1.86; 4.02)	0.472		1.21 (−1.04; 3.47)	0.290		1.19 (−1.63; 4.01)	0.408
*e.* *Very Unsafe*	47 (12.88)		5.24 (1.37; 9.11) **	0.008		3.29 (0.32; 6.26) *	0.030		5.55 (1.83; 9.27) **	0.003
3. Perception of Current Social Life										
*a.* *Very Satisfied*	15 (4.11)		−10.17 (−16.13; −4.22) ***	<0.001		−3.47 (−8.12; 1.17)	0.142		−7.28 (−13.07; −1.48) *	0.014
*b.* *Satisfied*	115 (31.51)	0.074	−5.98 (−8.52; −3.43) ***	<0.001	0.036	−3.56 (−5.54; −1.57) ***	<0.001	0.053	−5.12 (−7.59; −2.64) ***	<0.001
*c.* *Least Satisfied*	235 (64.38)		Reference			Reference			Reference	
4. Perception of Academic Performance										
*a.* *Very Satisfied*	18 (4.93)		−3.86 (−9.61; 1.88)	0.187		0.13 (−4.27; 4.52)	0.955		(−4.49; 6.64)	0.705
*b.* *Satisfied*	196 (53.70)	0.015	−2.86 (−5.35; −0.36) *	0.025	0.011	−1.90 (−3.81; 0.01)	0.050	0.003	(−3.52; 1.32)	0.371
*c.* *Least Satisfied*	151 (41.37)		Reference			Reference			Reference	
5. Concerned about Impairment of Study										
*a.* *High*	213 (58.36)		Reference			Reference			Reference	
*b.* *Medium*	126 (34.52)	0.004	−0.93 (−3.54; 1.67)	0.481	0.006	−0.02 (−2.01; 1.97)	0.986	0.002	−0.58 (−3.09; 1.93)	0.648
*c.* *Low*	26 (7.12)		−2.62 (−7.43; 2.19)	0.285		−2.63 (−6.31; 1.046)	0.160		−1.67 (−6.31; 2.97)	0.480
6. Concerned about the Family Member’s Earnings										
*a.* *High*	133 (36.44)		Reference			Reference			Reference	
*b.* *Medium*	133 (36.44)	0.014	−3.19 (−6.01; −0.36) *	0.027	0.007	−0.71 (−2.88; 1.46)	0.522	0.007	−1.42 (−4.70; 0.76)	0.157
*c.* *Low*	99 (27.12)		−2.50 (−5.56; 0.56)	0.108		−1.90 (−4.25; 0.44)	0.112		−1.31 (−4.92; 0.99)	0.192

* *p* < 0.05; ** *p* < 0.01; *** *p* < 0.001; β^#^ = Beta.

**Table 5:** Association of university support with DASS 21 subscales.

		Depression			Anxiety			Stress		
Features	*n* (%)	R^2^	β^#^ (95% CI)	*p*	R^2^	β (95% CI)	*p*	R^2^	β (95% CI)	*p*
1. Subject Related to COVID-19										
*a.* *Yes*	125 (34.25)		−2.93 (−6.47; 0.61)	0.104		0.12 (−2.59; 2.83)	0.930		−0.63 (−4.04; 2.78)	0.717
*b.* *No*	175 (47.95)	0.008	−1.17 (−4.53; 2.19)	0.494	0.003	1.02 (1.56; 3.59)	0.437	0.002	0.57 (−2.68; 3.81)	0.732
*c.* *Maybe*	65 (17.81)		Reference			Reference			Reference	
2. Financial/Mental Support for COVID-19										
*a.* *Yes*	140 (38.36)	0.006	−1.93 (−4.42; 0.56)	0.128	0.013	−2.11 (−4.01; −0.22) *	0.029	0.001	−0.67 (−3.08; 1.73)	0.581
*b.* *No*	225 (61.64)		Reference			Reference			Reference	
3. Online Class										
*a.* *Yes*	308 (84.38)	0.000	0.54 (−2.8; 3.88)	0.752	0.006	1.92 (−0.62; 4.47)	0.139	0.000	−0.01 (−3.23; 3.21)	0.996
*b.* *No*	57 (15.62)		Reference			Reference			Reference	

* *p* < 0.05; β^#^ = Beta.

## Data Availability

The data presented in this study are available on request from the corresponding author.
